# The Lyophilization Process Maintains the Chemical and Biological Characteristics of Royal Jelly

**DOI:** 10.1155/2015/825068

**Published:** 2015-04-30

**Authors:** Andresa Piacezzi Nascimento, Larissa Ariana Roveroni Moraes, Nathália Ursoli Ferreira, Gabriela de Padua Moreno, Fernanda Grassi Mangolini Uahib, Edna Aparecida Barizon, Andresa Aparecida Berretta

**Affiliations:** ^1^Laboratório de Microbiologia, Apis Flora Industrial e Comercial Ltda., 14020-670 Ribeirão Preto, SP, Brazil; ^2^Laboratório de Pesquisa, Desenvolvimento e Inovação, Apis Flora Industrial e Comercial Ltda., 14020-670 Ribeirão Preto, SP, Brazil; ^3^Faculdade de Ciências Farmacêuticas de Ribeirão Preto, Universidade de São Paulo, 14040-903 Ribeirão Preto, SP, Brazil

## Abstract

The alternative use of natural products, like royal jelly (RJ), may be an important tool for the treatment of infections caused by antibiotic-resistant bacteria. RJ presents a large number of bioactive substances, including antimicrobial compounds. In this study, we carried out the chemical characterization of fresh and lyophilized RJ and investigated their antibacterial effects with the purpose of evaluating if the lyophilization process maintains the chemical and antibacterial properties of RJ. Furthermore, we evaluated the antibacterial efficacy of the main fatty acid found in RJ, the 10-hydroxy-2-decenoic acid (10H2DA). Chromatographic profile of the RJ samples showed similar fingerprints and the presence of 10H2DA in both samples. Furthermore, fresh and lyophilized RJ were effective against all bacteria evaluated; that is, the lyophilization process maintains the antibacterial activity of RJ and the chemical field of 10H2DA. The fatty acid 10H2DA exhibited a good antibacterial activity against *Streptococcus pneumoniae*. Therefore, it may be used as an alternative and complementary treatment for infections caused by antibiotic-resistant *S. pneumoniae*.

## 1. Introduction

Frequent occurrence of infections caused by bacteria resistant to antibacterial agents is a common problem in hospitals. Resistant strains of bacteria are not inhibited or killed by the antibacterial agents at concentrations of the drugs achievable in the body after normal dosage. The resistance may increase the severity of disease and drive up health care costs. Therefore, the alternative use of natural products, like bee products, may be an important tool for the treatment of these infections.

Royal jelly (RJ) is a bee product widely used in traditional Oriental medicine. It is secreted from the mandibular and hypopharyngeal glands of worker honeybees (*Apis mellifera*) [1] and used to feed young larvae (from one to three days old), the queen bee larva, and the adult queen bee. RJ is involved in the sexual determination of the queen, besides its longevity.

In its composition, RJ contains proteins (approximately 50% of its dry mass), free amino acids, vitamins, sugars, fatty acids, sterols, and minerals [2–4]. Therefore, it is used worldwide as a functional food.

RJ also presents several pharmacological properties, such as antibacterial [3, 5, 6], antifungal [3, 7], antihypertensive [8], and estrogenic [9, 10] activities. Furthermore, RJ can play a significant role against colitis [11], induces mineralization in osteoblasts [10], improves erythropoiesis, glucose tolerance, and mental health [12], and may have antiatherogenic activity [13] and be beneficial in controlling diabetes outcomes [14]. Due to its pharmacological properties, RJ is used to supplement the treatment of several diseases, in many countries.

A large number of bioactive substances are present in RJ, such as antimicrobial peptides (royalisin and jelleins) [15–19], peptides with antihypertensive activity [8], and sterols and fatty acids with estrogenic effects [9], like the 10-hydroxy-2-decenoic acid (10H2DA) [10].

In this study, the abbreviation 10H2DA was used in order to differentiate 10H2DA from the other fatty acid present in RJ, the 10-hydroxydecanoic acid (10HDA), which is the saturated counterpart of 10H2DA.

Fresh RJ contains approximately 66% of water. Therefore, it is perishable and must be kept refrigerated to retain its nutritional value. Besides fresh RJ, the lyophilized one also is commercially available. Lyophilization process removes the water from RJ and is carried out by means of sublimation of the water (transition directly from the solid to the gaseous state). The advantage of the lyophilized RJ is that it can be stored at room temperature. Furthermore, it is usually sold in capsules in order to facilitate its use.

In the present study, we carried out the chemical characterization of fresh and lyophilized RJ and investigated their antibacterial effects, with the purpose of evaluating if the lyophilization process maintains the chemical and antibacterial properties of RJ. Furthermore, we evaluated the antibacterial efficacy of the 10H2DA.

## 2. Materials and Methods

### 2.1. Chemicals

Fresh RJ was purchased from Apis Nativa Produtos Naturais (Araranguá, SC, Brazil). Lyophilized RJ was obtained after lyophilization process of the fresh one, using a lyophilizator (Terroni, São Carlos, SP, Brazil). 10H2DA was purchased from Chromadex (Irvine, California, USA). Methanol HPLC grade was obtained from J.T. Baker. Water was treated in Milli-Q water purification system. The following culture media were used: Mueller Hinton agar and Mueller Hinton broth, which were purchased from Difco (Detroit, MI, USA); Mueller Hinton agar with 5% sheep blood (Plast Labor, Rio de Janeiro, RJ, Brazil); and Mueller Hinton broth supplemented with 5% lysed horse blood (Ebefarma Biológica e Agropecuária, Cachoeiras de Macacu, RJ, Brazil).

### 2.2. Chemical Characterization of RJ

Fresh and lyophilized RJ were analyzed by high-performance liquid chromatography (HPLC), using a Shimadzu apparatus equipped with a CBM-20A controller, a LC-20AT quaternary pump, a SPD-M 20A diode-array detector, and Shimadzu LC solution software, version 1.21 SP1. A Shimadzu Shim-Pack CLC-ODS (M) column (4.6 × 250 mm, particle diameter of 5 *μ*m, pore diameter of 100 Å) was used. The mobile phase consisted of methanol in pump B and of a solution of water-phosphoric acid (0.02% v/v), pH 2.5, in pump D. The mixture was eluted using an isocratic elution with 50% B and 50% D over a period of 22 min at a flow-rate of 0.8 mL/min. Detection was set at 215 nm.

RJ was dissolved with 5 mL of methanol (HPLC grade) in 10 mL volumetric flasks, subjected to sonication for 10 min and diluted to volume with Milli-Q water. The samples were filtered through a 45 *μ*m filter before analysis.

### 2.3. Antibacterial Activity

The following bacteria were used:* Staphylococcus aureus* ATCC 25923,* Staphylococcus aureus* ATCC 43300,* Staphylococcus epidermidis* ATCC 14990,* Streptococcus pneumoniae* ATCC 49619,* Escherichia coli* ATCC 25922,* Klebsiella pneumoniae* ATCC 10031,* Proteus mirabilis* ATCC 12453,* Salmonella enteritidis* ATCC 13076, and* Pseudomonas aeruginosa* ATCC 27853.

The broth microdilution method [20] was used to test the antibacterial activity of the samples. Mueller Hinton broth was used in the test with most of the bacteria. For* S. pneumoniae* Mueller Hinton broth supplemented with 5% lysed horse blood was used. The final RJ concentrations in relation to the dry weight ranged from 0.02 to 6.19% w/v. The final 10H2DA concentrations ranged from 7.81 to 250 *μ*g/mL.

The experiments were replicated three times for each bacterium.

### 2.4. Statistical Analysis

The data of the chemical characterization and antibacterial activity of the samples were submitted to two-way ANOVA. The data of the comparison of the bacteria were submitted to the one-way ANOVA and Bonferroni's Multiple Comparison Test. The established significance level was 5%. Statistical analysis of data was performed using the software Graph Pad Prism 5.

## 3. Results

### 3.1. Chemical Characterization of RJ

**Figure 1:**
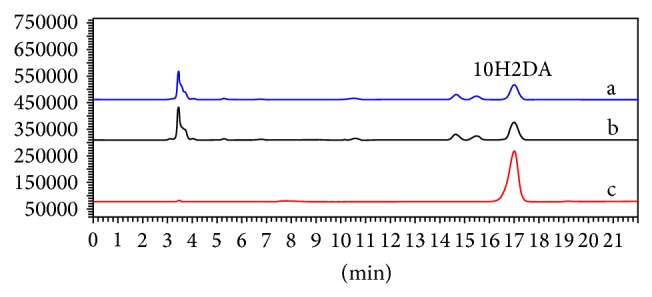
Chromatographic profile of the 10-hydroxy-2-decenoic acid (10H2DA) standard and of the lyophilized and fresh royal jelly (RJ). a: lyophilized RJ; b: fresh RJ; c: 10H2DA standard. The chromatograms were plotted at 215 nm, using HPLC, Shim-Pack CLC-ODS (M) column, and an isocratic elution with 50% methanol and 50% solution of water-phosphoric acid (0.02% v/v) over a period of 22 min at a flow-rate of 0.8 mL/min.

**Figure 2:**
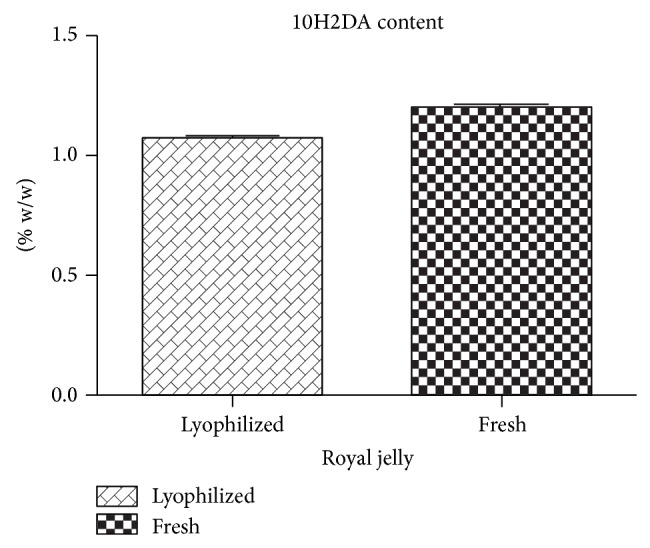
Content of 10-hydroxy-2-decenoic acid (10H2DA) (% w/w in relation to the dry weight) in fresh and lyophilized royal jelly.

The moisture contents of the lyophilized and fresh RJ were 0.96 and 69.21%, respectively. Chromatographic profile of the RJ samples showed similar fingerprints and the presence of 10H2DA in both samples (Figure 1). Furthermore, there was no significant difference between them (*P* > 0.05) (Figure 2).

### 3.2. Antibacterial Activity

Fresh and lyophilized RJ showed* in vitro* antibacterial activity against all bacteria evaluated (Table 1). There was no significant difference between them (*P* > 0.05). Results of MIC of the RJ samples were similar for most of the microorganisms, except* P. aeruginosa* and* S. pneumoniae*. The last one was the most susceptible microorganism to the samples (*P* < 0.05).

The fatty acid 10H2DA was not efficacious against most of the bacteria tested (Table 2). However, it exhibited antibacterial activity against* S. pneumoniae*.

## 4. Discussion

10H2DA is the major component of the lipid fraction of RJ; however, its content varies according to geographical origin of the sample [21]. Furthermore, 10H2DA is a unique RJ component [3] and is characterized like a biomarker of this bee product. Therefore, its detection and quantification may be considered as an identity and quality indicator of the RJ. In this study, chromatographic profile of the lyophilized and fresh RJ showed similar fingerprint and the presence of 10H2DA in both samples, demonstrating that the lyophilization process does not degrade the fatty acid.

The concentration of 10H2DA in fresh RJ is variable around the world and values of 0.33–2.54% were found by Genç and Aslan [22], 1.26–2.25% by Zhou et al. [23], and 1.58–3.39% by Garcia-Amoedo and Almeida-Muradian [24]. Sabatini et al. [25] suggest that 10H2DA content should be at least 1.4% for fresh royal jelly to attend quality control parameters. However, the data presented in this study showed that despite the low quantities of 10H2DA in the sample evaluated, the antibacterial activity was maintained.

Both samples of RJ were effective against all bacteria tested. It is important to mention that the samples were not submitted to any extraction process; that is, integral RJ samples were used (fresh or lyophilized raw material). Furthermore, our findings show that the lyophilization process maintains the antibacterial activity of RJ. In an* in vivo* study, Kayashima et al. [1] also demonstrated that lyophilized RJ maintains its developmental and physiological bioactivity in the fruit fly* Drosophila melanogaster* (model animal to examine the effects of RJ in multicellular organisms).

Gram-positive (staphylococci and* S. pneumoniae*) and Gram-negative bacteria (*E. coli*,* K. pneumoniae*,* P. mirabilis*,* S. enteritidis*, and* P. aeruginosa*) were killed by both samples. Two strains of* S. aureus* (ATCC 25923 and ATCC 43300) were evaluated in this study and both were killed by the RJ samples, including the* S. aureus* ATCC 43300, which is a methicillin-resistant* S. aureus* (MRSA), that is, a multidrug-resistant strain.

*P. aeruginosa*, the most frequent isolate from the burn wound [26], also was studied. The tested strain (ATCC 27853) also was evaluated by Boukraa [5], which demonstrated the efficacy of RJ from Algeria against this bacterium.

Besides* P. aeruginosa*, other bacteria usually isolated from the burn wound were evaluated in this study:* K. pneumoniae*,* E. coli*, and staphylococci [26]. Since RJ was effective against these bacteria, it may be used as an alternative and complementary therapy in wound infections caused by antibiotic-resistant bacteria. Boukraâ et al. [6] also demonstrated the efficacy of RJ against strains of* S. aureus* and* E. coli*.

Bacteria cited above also have been isolated in chronic wounds, like that present in individuals with* diabetes mellitus*. Therefore, RJ may be used to supplement the treatment of these wounds [27, 28]. Siavash et al. [27] demonstrated that RJ dressing was an effective and safe method for treating diabetic foot ulcers besides standard treatments (infection control, offloading, vascular improvement, and debridement if required). Wounds were washed, cleaned with saline, treated with sterile 5% RJ, and covered with sterile gauze. Most ulcers completely healed in 41 days (mean duration). Some pharmacological properties of the RJ may have contributed to wounds healing, like the antimicrobial, anti-inflammatory, and vasodilative activities.

*S. pneumoniae* was the most susceptible microorganism to the RJ samples. The fatty acid 10H2DA also exhibited a good antibacterial activity against this bacterium. It was considered that if the 10H2DA displayed an MIC less than 100 *μ*g/mL, the antibacterial activity was good [29].* S. pneumoniae* is a common cause of pneumonia, sinusitis, otitis media, meningitis, and septicemia. Since antibiotic resistance is an increasing threat with the diseases caused by* S. pneumoniae*, RJ and/or its bioactive substances may be used to supplement the treatment of these diseases.

*Streptococcus mutans* (bacterium associated with dental caries) also is susceptible to 10H2DA, which decreases the adherence of the bacterium to the cell surfaces and prevents* gtfB* and* gtfC* expression (genes that encode glucosyltransferases, which are important in* S. mutans* colonization and pathogenesis) [30].

Melliou and Chinou [3] also demonstrated the antibacterial efficacy of 10H2DA isolated from RJ from Greece against* S. mutans*. Furthermore, 10H2DA and other fatty acid derivatives isolated from RJ were evaluated by the disk diffusion method against other microorganisms. The samples showed antifungal and antibacterial activity, including against* Streptococcus viridans*, an oral pathogen [3].

Besides fatty acids, the antibacterial activity of RJ has been attributed to antimicrobial peptides, such as royalisin and jelleins. Royalisin is a potent antimicrobial peptide which acts against Gram-positive bacteria but not against Gram-negative bacteria [19]. In Gram-positive bacteria, royalisin decreases bacterial cell hydrophobicity and induces the disruption and dysfunction of membranes and cell walls [19].

Shen et al. [19] evaluated a recombinant royalisin from the RJ of Asian honeybee* Apis cerana* and demonstrated its efficacy against the following Gram-positive bacteria:* Bacillus subtilis*,* Micrococcus flavus*,* S. aureus*, and* Clostridium tetani*. However, the recombinant royalisin was inefficacious against Gram-negative bacteria (*E. coli*,* Salmonella typhimurium*, and* Proteus vulgaris*) and fungus (*Aspergillus oryzae*,* Penicillium viridicatum*, and* Pichia pastoris*).

Jelleins are effective against Gram-positive and Gram-negative bacteria [16]. Furthermore, they present antifungal activity [16].

In conclusion, fresh and lyophilized RJ maintained their 10H2DA contents and were effective against all bacteria evaluated; that is, the lyophilization process maintains the chemical and antibacterial properties of RJ. The fatty acid 10H2DA exhibited a good antibacterial activity against* S. pneumoniae*. Therefore, it may be used as an alternative and complementary treatment for infections caused by antibiotic-resistant* S. pneumoniae*.

## Figures and Tables

**Table 1:** Minimum inhibitory concentration (MIC) of lyophilized and fresh royal jelly; values are mean ± SD obtained from analyses in triplicate.

Bacterium	Royal jelly	
	MIC (% w/v)^a^	
	Lyophilized	Fresh
*Staphylococcus aureus* ATCC 25923	0.78 ± 0.00	0.78 ± 0.00
*Staphylococcus aureus* ATCC 43300	0.78 ± 0.00	0.78 ± 0.00
*Staphylococcus epidermidis* ATCC 14990	0.78 ± 0.00	0.78 ± 0.00
*Streptococcus pneumoniae *ATCC 49619	0.05 ± 0.00	0.05 ± 0.00
*Escherichia coli *ATCC 25922	0.78 ± 0.00	0.78 ± 0.00
*Klebsiella pneumoniae *ATCC 10031	0.78 ± 0.00	0.78 ± 0.00
*Proteus mirabilis *ATCC 12453	0.78 ± 0.00	0.78 ± 0.00
*Salmonella enteritidis *ATCC 13076	0.78 ± 0.00	0.78 ± 0.00
*Pseudomonas aeruginosa* ATCC 27853	1.55 ± 0.00	1.55 ± 0.00

a: % w/v in relation to the dry weight.

**Table 2:** Minimum inhibitory concentration (MIC) of 10-hydroxy-2-decenoic acid (10H2DA); values are mean ± SD obtained from analyses in triplicate.

Bacterium	10H2DA
	MIC (*μ*g/mL)
*Staphylococcus aureus* ATCC 25923	>250 ± 0.00
*Staphylococcus aureus* ATCC 43300	>250 ± 0.00
*Staphylococcus epidermidis* ATCC 14990	>250 ± 0.00
*Streptococcus pneumoniae *ATCC 49619	62.5 ± 0.00
*Escherichia coli *ATCC 25922	>250 ± 0.00
*Klebsiella pneumoniae *ATCC 10031	>250 ± 0.00
*Proteus mirabilis *ATCC 12453	>250 ± 0.00
*Salmonella enteritidis *ATCC 13076	>250 ± 0.00
*Pseudomonas aeruginosa* ATCC 27853	>250 ± 0.00
